# Supplementary material for: Cognitive performance in distinct groups of children undergoing epilepsy surgery—a single-centre experience
Source: PeerJ. 2019 Oct 8;7:e7790. doi: 10.7717/peerj.7790 (PMC6788437; doi:10.7717/peerj.7790)

**Trends in cognitive performance in the periods of pre-2011 vs. post-2011**

**Presurgical (the complete cohort)**

Source SS  df  MS   F Prob>F

------------------------------------------------

Groups  98.7 1 98.721   0.25 0.6188

Error 75122.6   189 397.474

Total 75221.3   190

**Postsurgical (the complete cohort)**

Source SS  df  MS   F Prob>F

-----------------------------------------------

Groups  91.7 1 91.662   0.2 0.654

Error 69971.6   154 454.361

Total 70063.3   155

**IQ/DQ change (the complete cohort)**

Source SS  df  MS   F Prob>F

------------------------------------------------

Groups   8   1 7.99 0.08   0.7747

Error 14766.6   152 97.1489

Total 14774.6   153

**
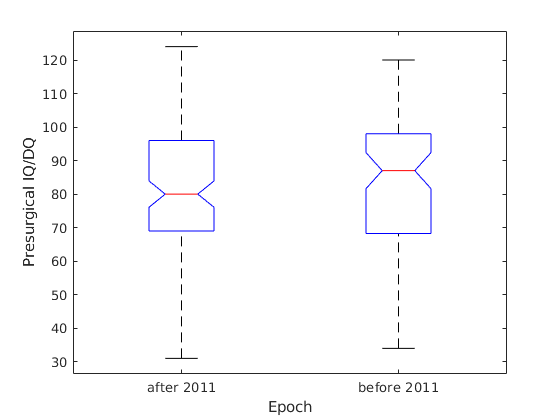
**

**
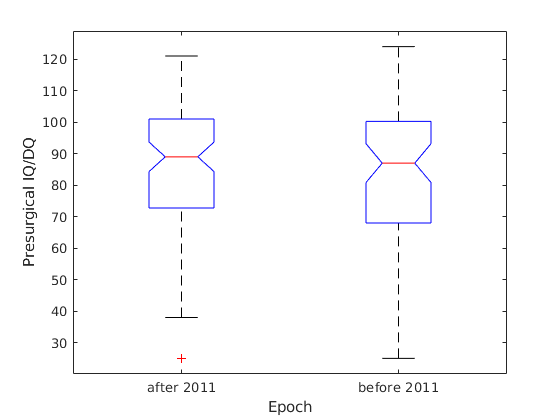
**

Postsurgical IQ/DQ

**
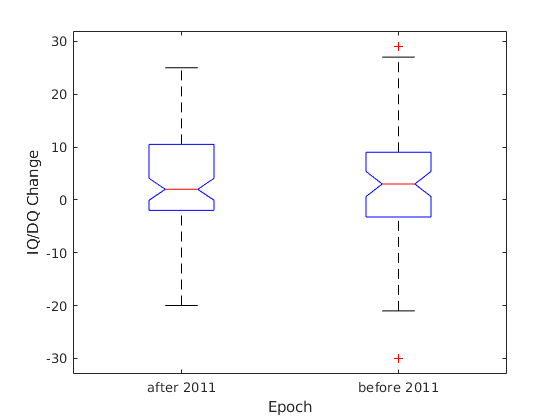
**

>> summary

TLE    93

XTLE   94

HEMI   16

**TLE presurgical**

Source   SS   df MS    F Prob>F

------------------------------------------------

Groups 319.97 1   319.965   0.89 0.3472

Error 31165.99   87 358.23

Total 31485.96   88

**TLE postsurgical**

Source SS   df MS    F Prob>F

-----------------------------------------------

Groups   18.2 1 18.219   0.05 0.8227

Error 25587.9   71 360.393

Total 25606.1   72

**TLE change**

Source SS   df MS    F Prob>F

-----------------------------------------------

Groups   7.8 1 7.8028   0.09 0.7691

Error 6287.47   70 89.8211

Total 6295.28   71


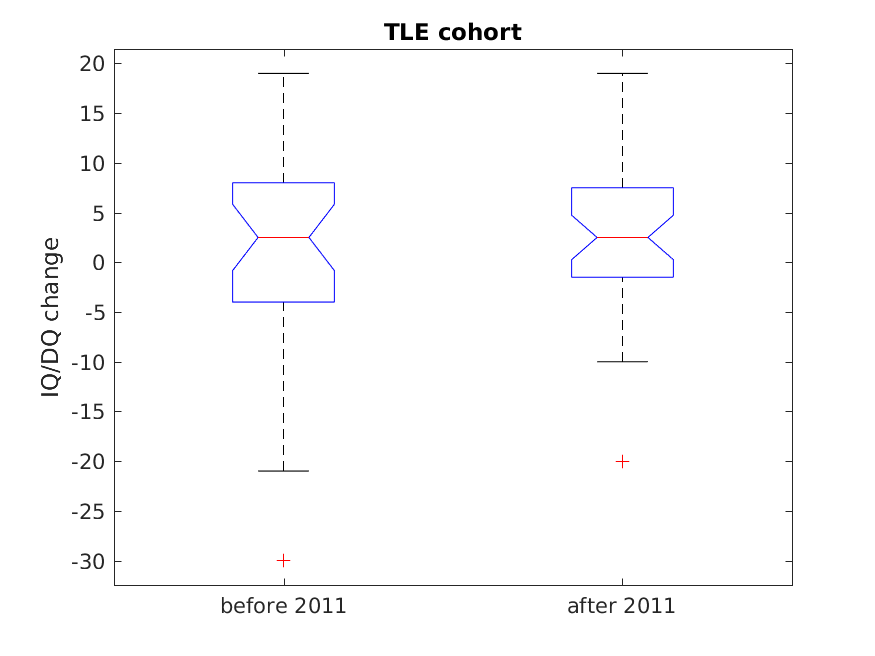

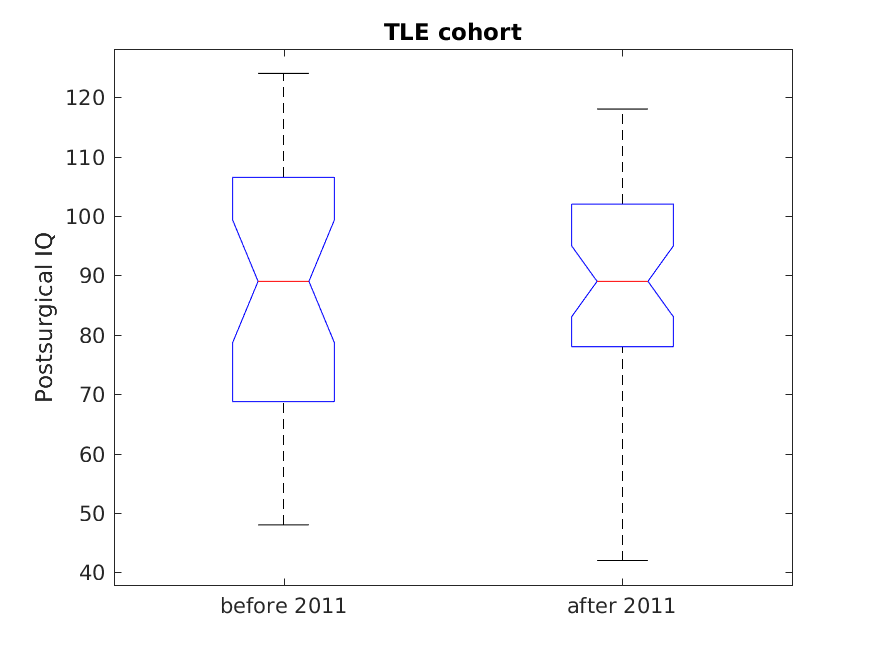

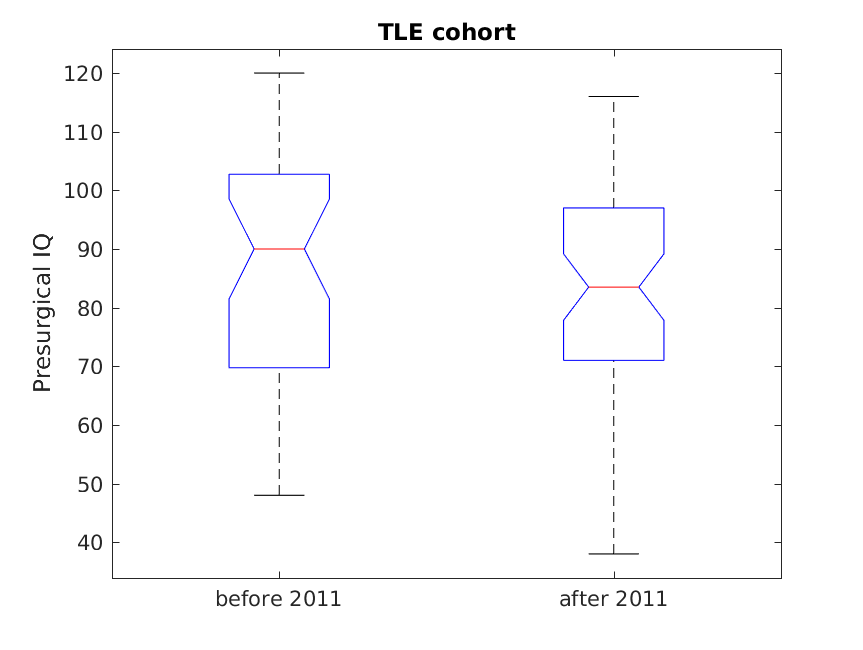
 **XTLE presurgical**

Source SS   df MS    F Prob>F

-----------------------------------------------

Groups 128.6 1   128.595   0.36 0.5485

Error 30839.5   87 354.476

Total 30968 88

**XTLE Postsurgical**

Source SS   df MS    F Prob>F

-----------------------------------------------

Groups 243.4 1   243.388   0.47 0.4969

Error 37586.1   72 522.029

Total 37829.5   73

**XTLE IQ/DQ Change**

Source SS   df MS    F Prob>F

-----------------------------------------------

Groups   0.78 1 0.7841   0.01 0.9282

Error 6800.89   71 95.7871

Total 6801.67   72


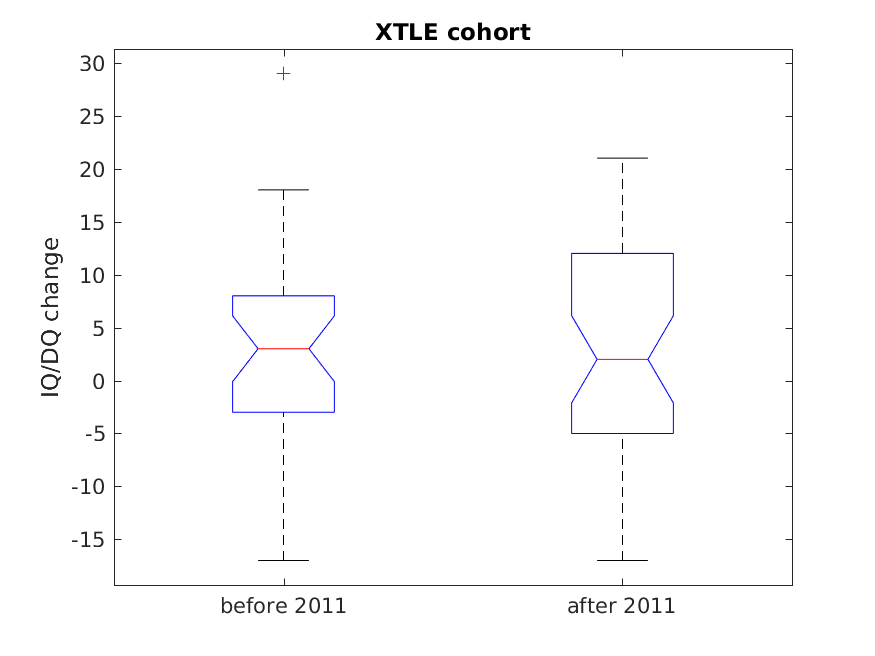

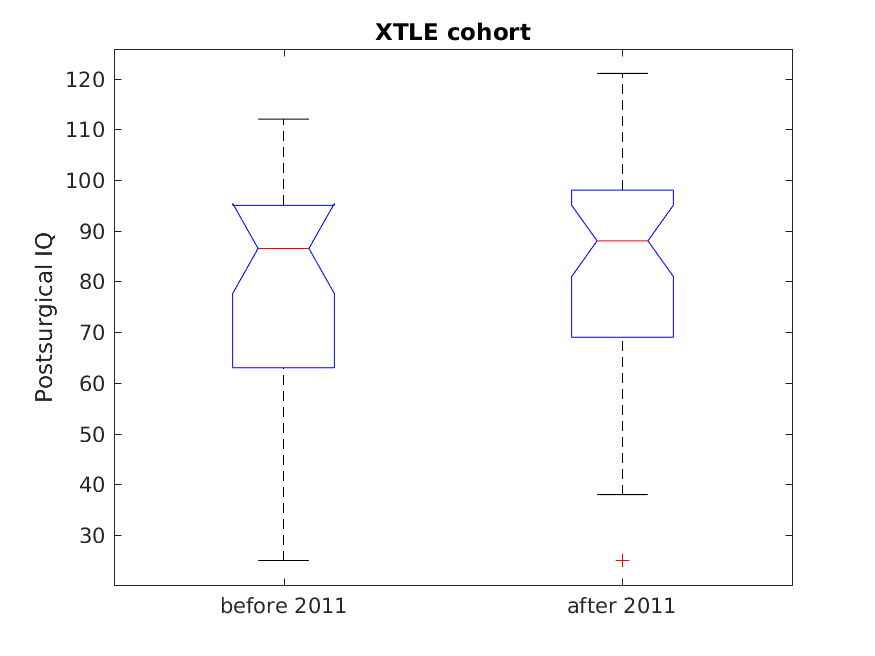

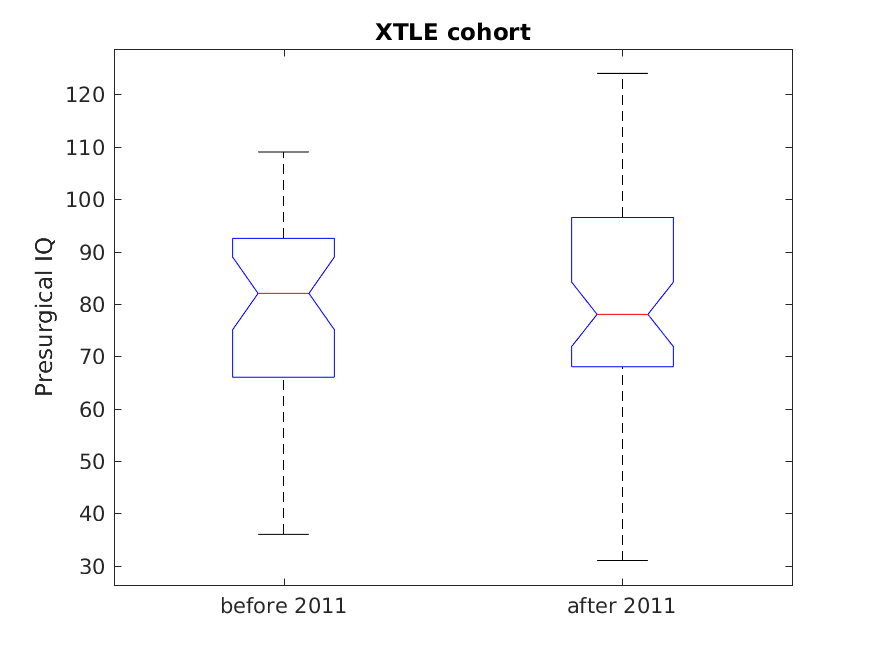


**HEMI presurgical**

Source SS   df MS    F Prob>F

-----------------------------------------------

Groups 344.74 1   344.741   0.52 0.4869

Error 7326.18   11 666.017

Total 7670.92   12

**HEMI Postsurgical**

Source SS df MS   F Prob>F

--------------------------------------------

Groups 283.5   1 283.5   0.48 0.5117

Error 4154.5   7 593.5

Total 4438 8

**HEMI IQ/DQ Change**

Source SS   df MS    F Prob>F

-----------------------------------------------

Groups 90.865   1 90.8651   1.62 0.2441

Error 393.357   7 56.1939

Total 484.222   8         
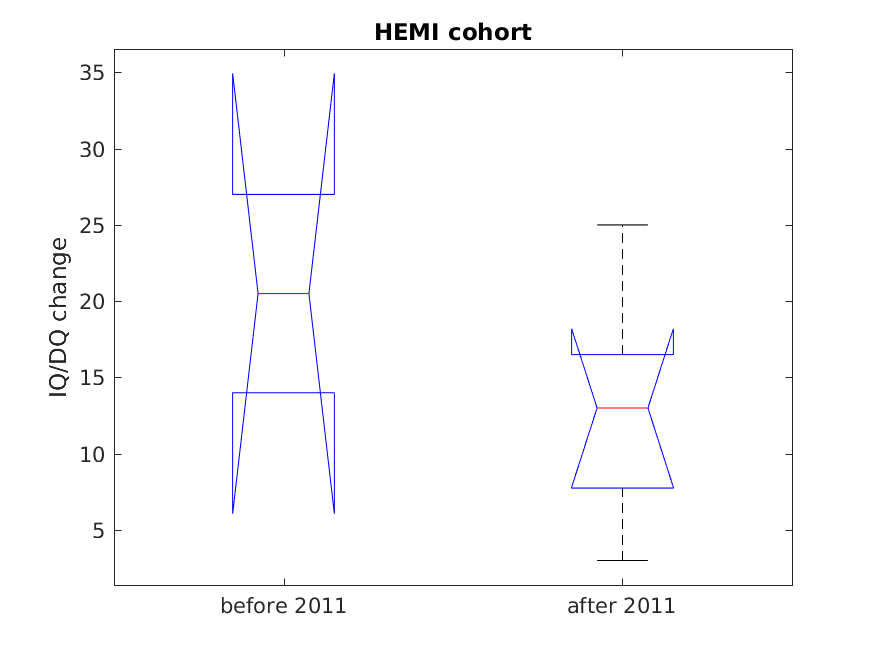

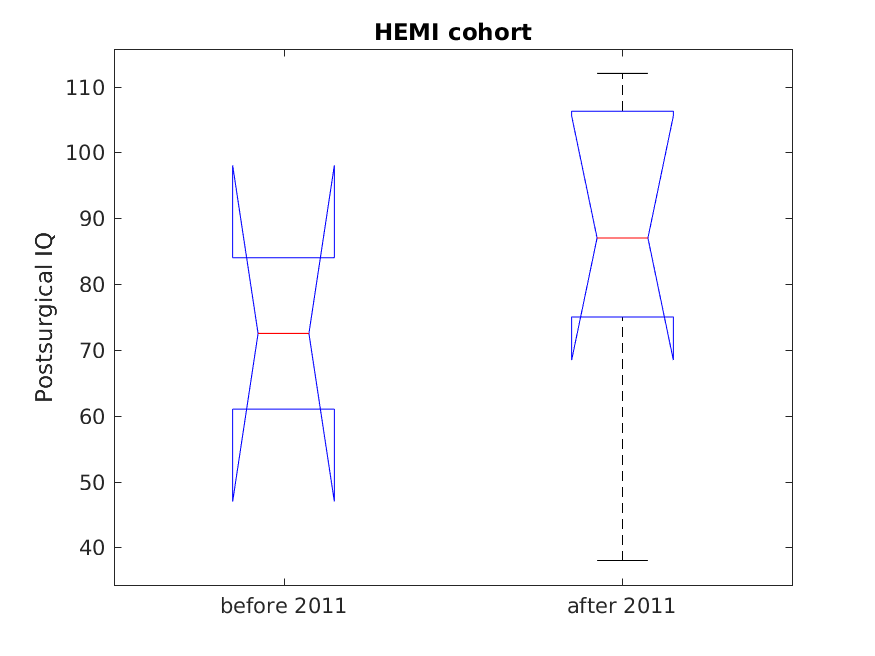

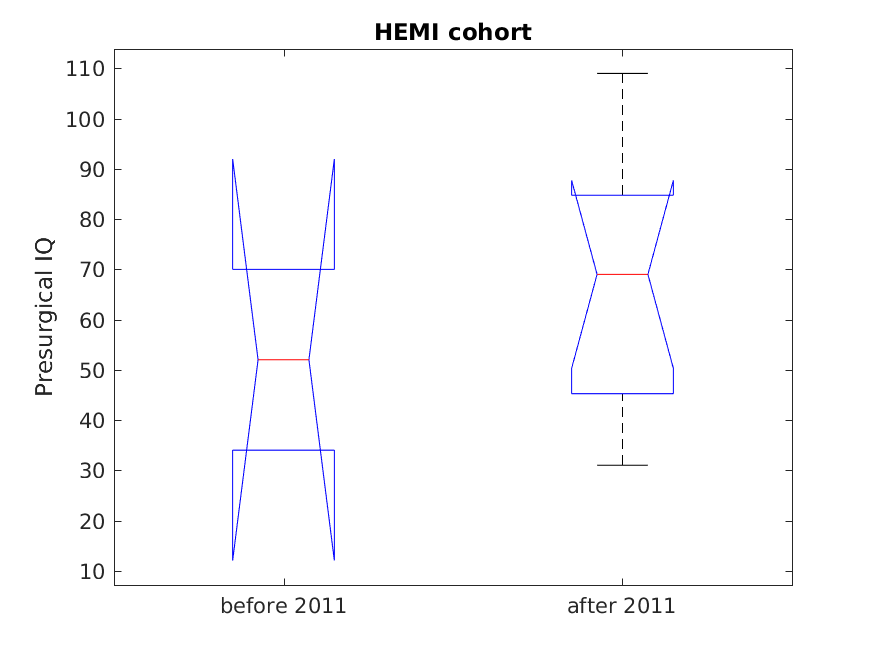


**Acquired vs. congenital lesions (comparison between the periods)**

>> summary

acquired       19

congenital    153

<undefined>     3

**Congenital Presurgical**

Source SS   df   MS    F Prob>F

------------------------------------------------

Groups   53.2 1 53.22 0.14   0.7103

Error 54163.4   141 384.138

Total 54216.7   142

**Congenital Postsurgical**

Source SS   df   MS    F Prob>F

------------------------------------------------

Groups    9.4 1 9.392   0.02 0.8903

Error 56995.7   116 491.342

Total 57005.1   117

**Congenital IQ/DQ Change**

Source SS   df   MS    F Prob>F

------------------------------------------------

Groups   10    1   10.0188   0.11 0.7428

Error 10556.4   114 92.6003

Total 10566.4   115


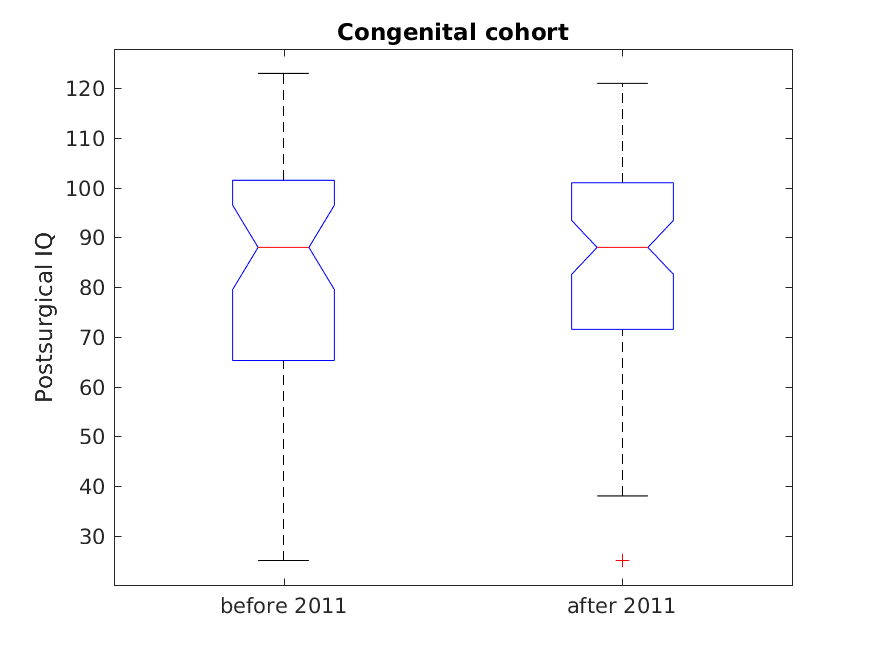


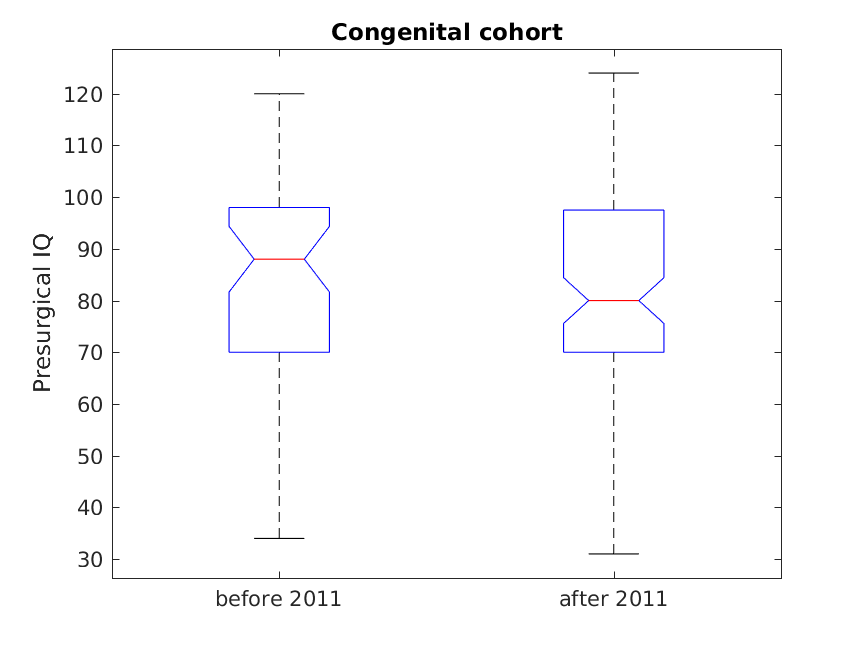


**Acquired cohort**

**Acquired Presurgical**

Source SS   df MS    F Prob>F

----------------------------------------------

Groups 365.76 1   365.76 0.7   0.4154

Error 8371.35   16 523.209

Total 8737.11   17

**Acquired Postsurgical**

Source SS   df MS    F Prob>F

-----------------------------------------------

Groups 13.15 1 13.149   0.03 0.8581

Error 4731.21   12 394.267

Total 4744.36   13

**Acquired IQ/DQ Change**Source SS   df MS    F Prob>F

-----------------------------------------------

Groups 56.01 1 56.006   0.49 0.4957

Error 1361.21   12 113.434

Total 1417.21   13


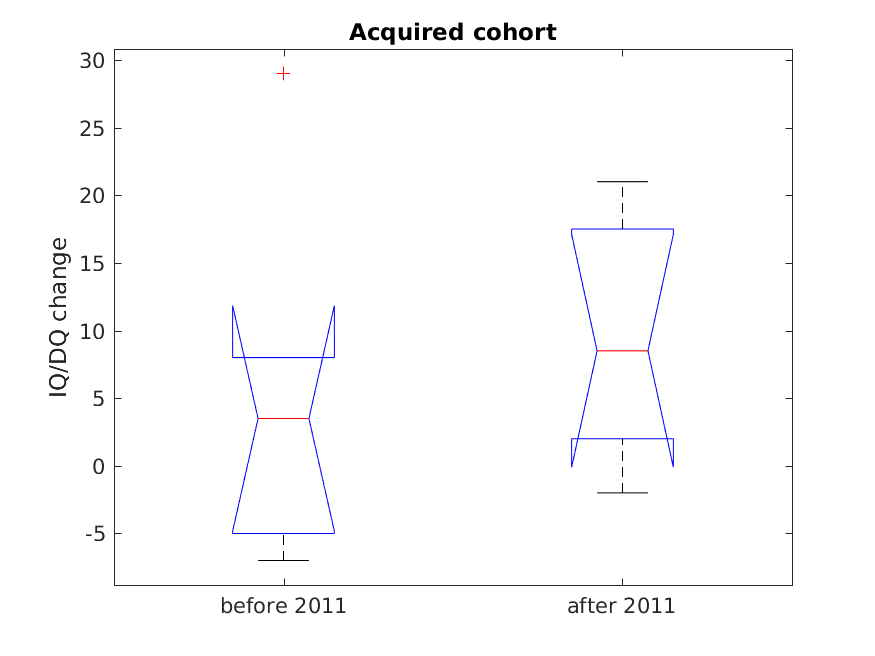

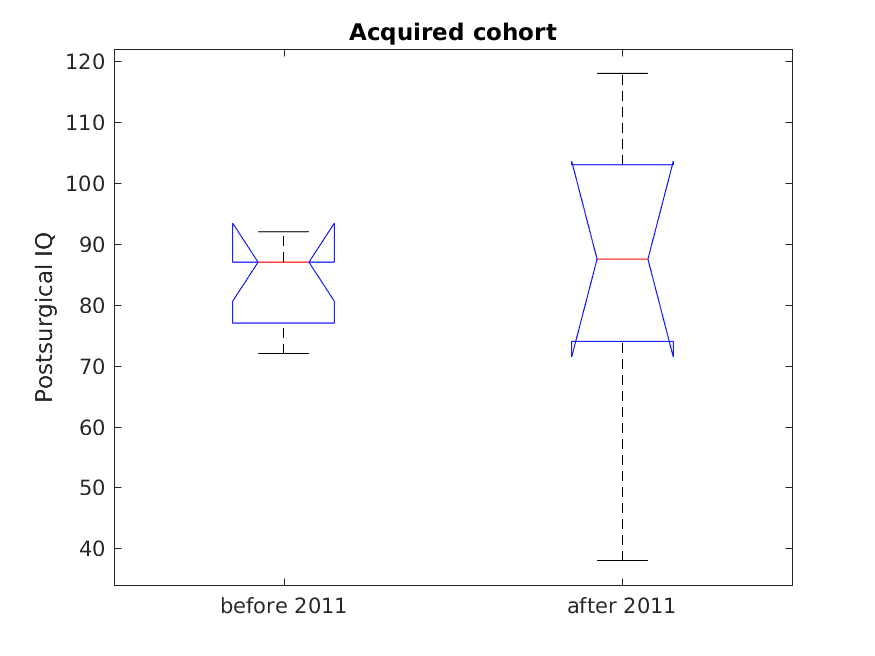
Presu


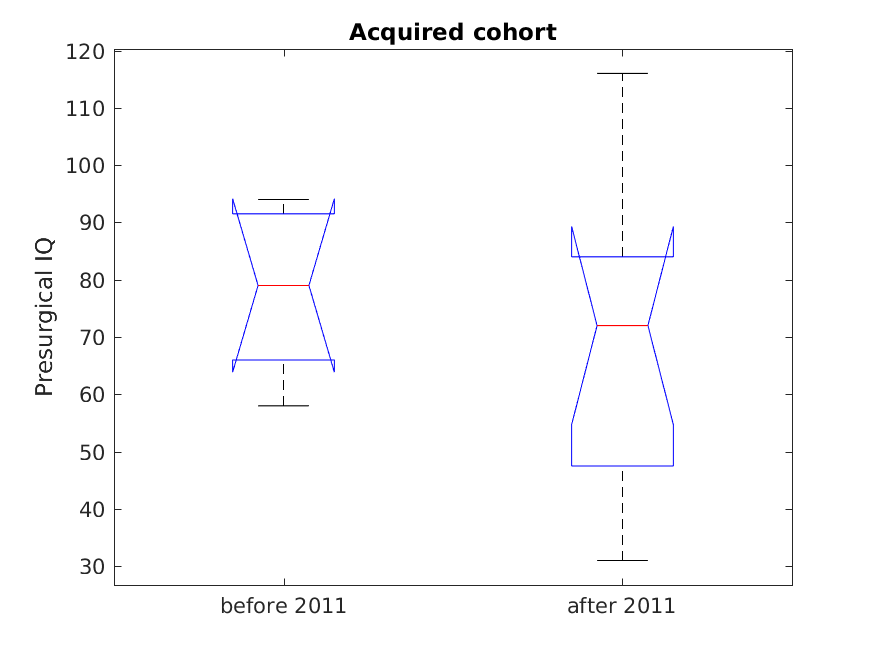


**Acquired vs. congenital lesions (over the entire follow-up period)**

>> summary

acquired       19

congenital    149

<undefined>     3

**Presurgical**

Source SS   df   MS    F Prob>F

------------------------------------------------

Groups 1413.8 1   1413.81   3.52 0.0624

Error 62596.9   156 401.26

Total 64010.7   157

**Postsurgical**

Source SS   df   MS    F Prob>F

------------------------------------------------

Groups    2.5 1 2.489   0.01 0.943

Error 61516.1   127 484.379

Total 61518.6   128

**IQ/DQ Change:**

Source SS   df   MS   F Prob>F

-----------------------------------------------

Groups 331.2 1   331.17   3.49 0.0643

Error 11877.4   125 95.02

Total 12208.6   126


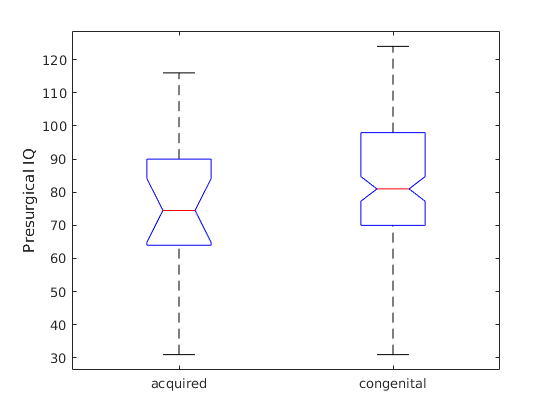

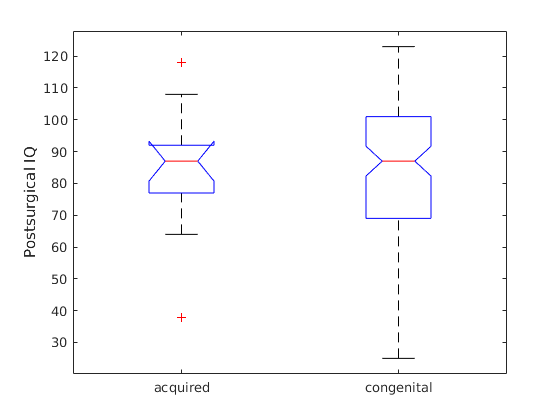

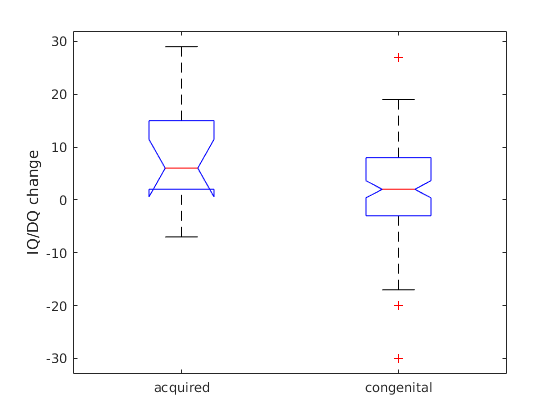

Supplement: Supplemental Information 1 — FCD: focal cortical dysplasia, MCD: malformations of cortical development, TSC: tuberous sclerosis complex, TLE: temporal lobe epilepsy, XTLE: extratemporal lobe epilepsy, HEMI: hemispheric epilepsy. [file peerj-07-7790-s001.docx]
